# Measuring Hand Function in Older Adults: The Need for Better Assessment Tools

**DOI:** 10.1093/geroni/igaa057.653

**Published:** 2020-12-16

**Authors:** Rachel Logue, Elana Goldenkoff, Michael Vesia, Susan Brown

**Affiliations:** 1 University of Michigan, Ann Arbor, Michigan, United States; 2 University of Michiagn, Ann Arbor, Michigan, United States

## Abstract

Aging is associated with a decline in hand muscle strength, dexterity, and tactile perception, leading to difficulties in activities of daily living and reduced independence (Millan-Calenti et al., 2010). However, current assessments do not adequately capture sensorimotor skills that underlie everyday activities such as dressing and food preparation. This study examined the ability of two novel assessment devices to detect age-related changes in hand force control and tactile pattern discrimination. Sensorimotor function was assessed in 13 healthy older adults (mean age 72.2 +/- 5.5y) and 13 young adults (mean age 20 +/- 1.4y). Maximum grip force (MVC), tactile sensation, and hand dexterity were measured using standard clinical techniques. Novel assessments consisted of submaximal (5-20% MVC) grip force tracking and computer-controlled tactile pattern recognition. Monofilament testing of tactile sensation was normal in the older group. In contrast, both the accuracy and speed associated with identifying tactile patterns was significantly worse in older (p<0.001) compared to young adults for both hands. While maximum grip force was similar in both groups, the ability to smoothly produce (p<0.05) and maintain (p<0.02) low grip force levels was compromised in older adults. Manual dexterity (Grooved Pegboard test) was significantly reduced in the older group (p<0.001) regardless of hand. These results indicate that the ability to extract meaningful information from tactile feedback and control low levels of force - aspects of fine hand control associated with activities of daily living – are impaired in older adults and underscore the need for more sensitive measures of hand function.

